# Occupational factors related to burnout among agents of a service center of a telecommunications operator in Tunisia

**DOI:** 10.1192/j.eurpsy.2023.1826

**Published:** 2023-07-19

**Authors:** Z. Athimni, A. Chouchen, H. Kalboussi, M. T. Haloul, A. Aloui, A. Gaddour, M. Bouhoula, M. O. Maoua, A. Brahem, O. Maalel, S. Chatti, I. Kacem, N. Mrizak

**Affiliations:** 1 Service de médecine du travail et de pathologies professionnelles - CHU Farhat Hached 4002 Sousse, Tunisie., Université de sousse; 2 Département de médecine de famille, Faculté de médecine Ibn El Jazzar, Université de Sousse, Tunisie., Sousse; 3 Service de médecine du travail, Hôpital Régional Ibn El Jazzar Kairouan, Tunisie, Université de sousse, Kairouan, Tunisia

## Abstract

**Introduction:**

Burnout syndrome (BO), considered to be the final stage of stress, is a dynamic process resulting from the gradual loss of the employee’s ability to face psychosocial risk factors and the exhaustion of personal resources. This syndrome can affect all professional categories. It represents a major threat for the worker and has a high economic cost

**Objectives:**

To identify the professional factor associated with BO among agents of a service center of a telecommunications operator in the governorate of Sousse in Tunisia

**Methods:**

Cross-sectional descriptive study, conducted from February 1^st^, 2020 to January 31^st^, 2021 among agents of a service center of a telecommunications operator in the agencies of the governorate of Sousse. The collection of data was based on an anonymous self-questionnaire. The evaluation of stress was done via validated measurement instruments, namely the questionnaires of Siegrist and Karasek and the evaluation of BO by the Maslach Burnout Inventory (MBI).

**Results:**

Ninety four actel agents were identified in our study, and 59 participants answered the questionnaire. The average age of our population was 43.93 ± 8 years. The female gender was predominant with a sex ratio of 0.48. The Karasek Job Content Questionnaire showed that 23.7% of the participants were in job strain. Furthermore, 28.3% of our total population had an imbalance between extrinsic efforts and rewards. The assessment of burnout by the Maslach Burnout Inventory showed that 64.2% of the workers were in occupational BO, and in 11.8% of these cases, BO was considered high. Our study showed that work-related organisational and environmental factors were significantly associated with BO, such as the number of customers seen per day: exceeding 50 (p = 0.048) and being a victim of verbal aggression (p = 0.038).

**Conclusions:**

The results of this study showed that BO among agents of a service center of a telecommunications operator in Tunisia, was significantly associated with some professional factors. Therefor, in the future, it would be advisable to improve the working conditions of these agents by introducing collective and organisational preventive measures (primary prevention) and individual measures (secondary prevention) and by facilitating their professional reintegration (tertiary prevention) to avoid relapses.

**Disclosure of Interest:**

None Declared

